# Spatial–temporal evolution and peer effects of urban green development efficiency in China

**DOI:** 10.1038/s41598-024-58591-w

**Published:** 2024-04-11

**Authors:** Jialiang Zhou, Mingchun Zhong

**Affiliations:** School of Economics and Trade, Fujian Jiangxia University, Fuzhou, 350108 China

**Keywords:** Ecology, Environmental sciences

## Abstract

In the process of global urban development, there are urgent ecological security and environmental pollution problems, green development is the fundamental way for urban sustainable development, economic transformation and mitigation of ecological and environmental problems. Based on the panel data of 283 cities at prefecture level and above in China from 2003 to 2017, this paper analyzes spatial–temporal evolution characteristics of urban green development efficiency (UGDE) and the peer effects of UGDE between cities of different grades. It is found that during the study period, in terms of temporal evolution, the average UGDE in China increased from 0.47 in 2003 to 0.61 in 2017, with a cumulative growth rate of 29.79%, showing a rising trend in general. In terms of spatial evolution, the number of low-efficiency cities and medium-efficiency cities continued to decrease. The eastern region has always been the main distribution area of higher-efficiency cities and high-efficiency cities; in the central region, UGDE in most cities improved significantly; in the western region, UGDE has always lagged behind that in the eastern and central regions. In addition, the center of gravity of UGDE presented a trend of northwest migration in general, with a total displacement of 100.07 km, and UGDE showed a spatial dispersion trend. The empirical results indicate that the improvement of UGDE in large cities has a driving effect on that in neighboring medium cities and small cities through the positive peer effect, and the growth of UGDE in medium cities has a promoting effect on that in neighboring small cities through the positive peer effect; the increase of UGDE in medium cities has a positive peer effect on that in neighboring large cities, and the growth of UGDE in small cities has a positive peer effect on that in neighboring medium cities; UGDE promotes each other between large cities through the positive peer effect.

## Introduction

In recent years, with the acceleration of global urbanization and industrialization, the emissions of greenhouse gases, mainly carbon dioxide, have increased sharply, and the problem of environmental pollution has become increasingly prominent, causing serious environmental damage and incalculable economic losses to human society. In fact, the ecological environmental problem has become an important challenge facing all countries at present, which is related to the sustainable development of the global economy in the future ^1^. Based on this background, green development, as an effective way to solve the problems of economic transformation, ecological security and environmental pollution, is gradually becoming the consensus of all countries in the world, which has triggered the global collective action to promote green development. In 2022, the US government proposed up to $369 billion in "green subsidies" and related incentives in the newly introduced "Inflation Reduction Act" to promote the research, development and manufacturing of electric vehicle batteries and other green products. The European Union passed a legislative proposal to ban the sale of fossil fuel vehicles, and in early 2023 issued a "Green Deal Industrial Plan" to develop a green economy and promote the development of green industries. As the largest developing country in the world, China's economy has achieved remarkable achievements after more than 40 years of rapid growth, but China has paid a huge price in terms of the environment, which has aggravated the global environmental pollution. According to China’s Ecological Environment Status Bulletin in 2019, among 337 cities at the prefecture level and above in China, 180 cities exceeded the standard in the air quality pollution index, accounting for 53.41% of all cities, and the urban environmental pollution problem is serious. Under the constraints of existing resources and environment, green development is the inevitable way for China's economic transformation, which is not only conducive to alleviating China's ecological and environmental problems, but also of great significance for realizing the Sustainable Development Goals (SDGs) of the United Nations.

Urban green development efficiency (UGDE) is an important index to measure the quality of economic development and a core concept in the field of green development. Its connotation is to obtain the maximum economic benefits with the minimum cost of resources and environment, so as to realize the coordination between resources, environment and economic development, which provides a measurable scheme for the evaluation of urban green development. As the economic links between cities become closer and closer, the coordinated development of regional economy has become an important force to tap the potential of economic growth and promote urban development. It is undeniable that urban green development also needs to rely on regional coordinated development to promote, through the coordination of green development policies and measures among cities, give play to the spatial interaction between cities, and jointly promote urban green development. Therefore, this paper studies the peer effects of green development efficiency between cities of different grades, thus explaining the internal driving force of regional green coordinated development, which has important theoretical significance and practical value for the formulation and implementation of urban green development policies and the improvement of urban green development efficiency.

The peer effect originates from social network theory, which holds that information exchange between individuals will form a complex social relationship network, and individual behavior and decision making in society will be affected not only by their own characteristics, but also by group behaviors with similar characteristics associated with them, showing a tendency of consistency between individual and group behaviors^2–6^. In a large number of studies, the existence of peer effect in entrepreneurial intention^7,8^, personal consumption decision^6,9,10^, corporate strategic decision^11–13^ and other fields have been confirmed. From the perspective of social network, the set of interactions and influences between cities constitutes an urban economic network, and the cooperation and competition relationships in urban economic network lead to strategic interaction between cities, in essence, all kinds of cities in urban economic network belong to the same group. For example, Shi et al. found that there are the peer effects of the economic growth between cities of different income levels^14^. At present, promoting green development has become the main economic behavior of cities, due to the existence of competition and cooperation, there is strategic interaction between cities in terms of green development, and the green development efficiency of this city may affect the green development efficiency of neighboring cities, which has a peer effect.

The academic community carried out systematic research on UGDE. In the early stage of UGDE was put forward, scholars paid more attention to the measurement methods of UGDE and proposed different measurement methods, including dynamic-SBM (DSBM) model^15^, the data envelopment analysis game cross-efficiency mode^16^, DEA super-efficient unexpected output model^17^, super-efficiency undesirable SBM model^18–21^, Metafrontier Malmquist Luenberger index^22^, SBM model^23,24^. With the maturity of research on the measurement methods of UGDE, many scholars began to analyze the spatial–temporal evolution pattern and characteristics of UGDE. For example, based on a sample of Chinese cities, the existing literature found that UGDE has spatial agglomeration characteristic, the spatial evolution process of different kinds of cities is path-dependent, and there is spatial club convergence, that is, areas with high UGDE are concentrated and drive low UGDE elsewhere^20^. However, in Beijing-Tianjin-Hebei region and Shandong Province of China, UGDE shows spatial differentiation characteristic^18,19^. In the urban agglomerations of the upper reaches of the Yangtze River in China, the green development efficiency shows an evolutionary feature of "down-up-down"^25^, and has hierarchical structure differences among different urban agglomerations. In Xuzhou metropolitan area, China, the overall efficiency of green development presents a downward trend, and the spatial agglomeration narrows, with obvious regional differences^26^.

In recent years, the research on the factors affecting the efficiency of urban green development has become increasingly popular, and the relevant research results emerged in an endless stream. The scholars argued the impact of technological innovation, financial agglomeration, industrial structure adjustment, tourism development, producer services agglomeration, carbon emission rights, and digital economy on UGDE. It is found that technological innovation significantly improves UGDE^16^, and the impact of technological innovation on UGDE in eastern cities of China is greater than that in central and western cities of China, and the higher the administrative level of a city, the greater the impact of technological innovation on UGDE^27^. The development of digital technology, which belongs to one of the categories of technological innovation, significantly improves UGDE, its influence mechanism is to improve the intensity of environmental regulation and the level of technological innovation^28^. Financial agglomeration shows the periodic characteristics of diffusion and equilibrium, which have the non-equilibrium impact on UGDE^29,30^. In the process of industrial structure adjustment, the rationalization and upgrading of industrial structure significantly improve UGDE, and the upgrading of industrial structure has a greater impact on UGDE than the rationalization of industrial structure^21^. Under the exogenous impact of the pilot policy of "low-carbon city", the spatial spillover effect of tourism development on UGDE has U-shaped and spatial heterogeneity characteristic^31^. The professional agglomeration of producer services has a significantly positive effect on urban green development performance, while the effect of diversified agglomeration is opposite^32^. The carbon emission trading policy improves UGDE by adjusting energy structure, improving resource mismatch and promoting green technological innovation^17^. Digital economy plays a significant role in promoting UGDE, and the impact of digital economy on UGDE in eastern China and big cities is greater than that in central and western China and small cities^33^. With the deepening of research, existing literature explored the spatial spillover effect of UGDE and found that there was a significant positive effect^19,20^.

Through the review of the above literature, this paper finds that the relevant research on UGDE achieved considerable progress and relatively fruitful results in the aspects of measurement methods, spatial–temporal evolution pattern and characteristics, and influencing factors, which provides a rich reference basis for relevant departments to improve urban governance and enhance UGDE. In addition, the spatial spillover effect of UGDE has been confirmed^19,20^. However, the existing studies did not involve the peer effect caused by the strategic interaction between cities. The peer effect is essentially a spatial spillover effect, but its research focus is on the strategic interaction among economic agents, and the research perspective tends to be micro. Compared with the existing literature, the possible marginal contributions of this paper are as follows: Firstly, based on the analysis of the spatial–temporal evolution characteristics of UGDE, this paper constructs a spatial econometric model, divides Chinese cities into large cities, medium cities and small cities, examines the peer effects of UGDE between cities of different grades, explores the spatial interaction of UGDE between cities of different grades, and then explains the internal driving force of regional green coordinated development. Secondly, this paper divides Chinese cities into eastern cities, central cities and western cities according to geographical location, and analyzes the regional heterogeneity of peer effects of UGDE.

Based on the panel data of 283 cities at prefecture level and above in China from 2003 to 2017, firstly, this paper uses SE-SBM model with unexpected output to measure urban green development efficiency. Secondly, the spatial–temporal evolution characteristics of UGDE are analyzed based on the measurement results. Finally, this paper builds a spatial econometric model to analyze the peer effects of UGDE between cities of different grades. The rest of this paper is arranged as follows: The second part is the methodology and data; the third part is the result analysis; the fourth part is the discussion; the fifth part is the conclusion.

## Methodology and data

### SE-SBM model

The traditional DEA model, such as CCR model and BCC model, does not take the undesired outputs into account such as negative external effects on the environment caused by the production process, and ignores the slack problem of input–output variables, so it cannot accurately measure the efficiency value. In order to solve the slack problem of input–output factors, Tone proposed a slack-based measure (SBM) model^34^, which incorporates slack variables into the objective function, and is a non-radial and non-angular efficiency measurement method. However, this model cannot further decompose the effective decision-making units with an efficiency value of 1. Based on this, Tone built a super-efficiency SBM model (SE-SBM) including undesired outputs based on the original SBM model^35^. The model synthesizes the advantages of the super-efficiency model and the SBM model. On the one hand, the undesired output is incorporated into the model, on the other hand, in order to avoid the loss of effective decision-making units information, the effective decision-making units with an efficiency value of 1 is further decomposed. UGDE is an input–output relationship that takes environmental pollution into account in the process of urban development. Therefore, this paper draws on the research method of Tone and uses the SE-SBM model to measure UGDE^35^. The model is as follows:

Suppose there are *n* decision making units (DMUs), and each DUM consists of *m* inputs, *r*_*1*_ desired outputs, and *r*_*2*_ undesired outputs. The vector matrix of input elements is defined as *X* = [*x*_*1*_, …, *x*_*n*_] ∈ R^m×n^, the vector matrix of expected output is defined as *Y*^*g*^ = [*yg 1*, …, *yg n*] ∈ R^s1×n^, and the vector matrix of unexpected output is defined as *Y*^*b*^ = [*yb 1*, …, *yb n*] ∈ R^s2×n^, where *X* > *0*, *Y*^*g*^ > *0*, *Y*^*b*^ > *0*. Then the set of production possibilities under constant returns to scale is defined as:

1 $$ {\text{p}}\left( {x,y^{g} ,y^{{\text{b}}} } \right){ = }\left\{ {\left( {x,y^{g} ,y^{{\text{b}}} } \right)|x \ge \lambda X,y^{g} \le \lambda Y^{g} ,y^{{\text{b}}} \ge \lambda Y^{{\text{b}}} ,\lambda \ge 0} \right\} $$

Then the linear programming formula of the SE-SBM model is as follows:

2 $$ \begin{array}{*{20}l} {\rho = \min \frac{{1 + \frac{1}{m}\sum\nolimits_{i = 1}^{m} {\frac{{s_{i}^{ - } }}{{x_{ik} }}} }}{{1 - \frac{1}{{r_{1} + r_{2} }}\left[ {\sum\nolimits_{r = 1}^{{r_{1} }} {\frac{{s_{r}^{{g^{ + } }} }}{{y_{{_{rk} }}^{g} }}} + \sum\nolimits_{t = 1}^{{r_{2} }} {\frac{{s_{t}^{{b^{ - } }} }}{{y_{{_{tk} }}^{b} }}} } \right]}}} \hfill \\ {s.t.\sum\limits_{j = 1,j \ne k}^{n} {x_{ij} \lambda_{j} - s_{r}^{ - } \le x_{ik} } } \hfill \\ {\sum\limits_{j = 1,j \ne k}^{n} {x_{tj} \lambda_{j} + s_{r}^{{g^{ + } }} \ge y_{{_{rk} }}^{g} } } \hfill \\ {\sum\limits_{j = 1,j \ne k}^{n} {y_{{_{rj} }}^{b} - s_{t}^{{b^{ - } }} \le y_{{_{tk} }}^{b} } } \hfill \\ {1 - \frac{1}{{r_{1} + r_{2} }}\left[ {\sum\limits_{r = 1}^{{r_{1} }} {\frac{{s_{r}^{{g^{ + } }} }}{{y_{{_{rk} }}^{g} }}} + \sum\limits_{t = 1}^{{r_{2} }} {\frac{{s_{t}^{{b^{ - } }} }}{{y_{{_{tk} }}^{b} }}} } \right] \ge 0} \hfill \\ {s^{ - } > 0,s^{b} > 0,s^{g} > 0} \hfill \\ {i = 1,2, \ldots ,m;r = 1,2, \ldots ,q;j = 1,2, \ldots ,n\left( {j \ne k} \right)} \hfill \\ \end{array} $$

where *ρ* is the target efficiency value, that is, UGDE, *ρ* > 0, the larger the value of *ρ*, the higher the efficiency. *s*^*-*^,* s*^*g*^ and* s*^*b*^ represent the relaxation variables of input, expected output and unexpected output respectively; *λ* indicates the weight.

In terms of the selection of indicators to measure UGDE, input and output indicators are determined based on relevant studies^18,20^. This paper selects input indicators from three aspects, including capital, labor and resources. In terms of capital input, urban capital stock is used to represent capital input. Since capital stock data cannot be directly obtained, capital stock is calculated through the perpetual inventory method based on actual fixed asset investment^36^. In terms of labor input, the total number of urban employees is selected as the index of labor input, and it is the sum of the number of urban unit employees and the number of private and individual employees. In terms of resource input, this paper selects input indicators from three aspects, including water resources, energy and land resources, uses the total water supply as the input index of water resources, selects the total electricity consumption of the whole society as the energy input index, and uses the area of urban construction land to represent the input index of land resources, urban construction land refers to the urban land used for the construction of buildings and structures. In terms of desired output, the actual gross regional product is used as the expected output indicator. In terms of undesired output, this paper takes the environmental pollution in the process of urban development as the undesired output, specifically, the gross amount of industrial wastewater discharge, the gross amount of industrial smoke and dust discharge and the gross amount of industrial sulfur dioxide discharge are taken as the undesired output indicators.

### Spatial econometric model

In order to explore the peer effects of UGDE and analyze the spatial interaction between cities, that is, to investigate whether the change of UGDE in this city has an impact on that in neighboring cities, this paper introduces the spatial weight matrix *W* into the general econometric model, and establishes a spatial panel autoregressive model. The spatial autoregressive coefficient of this model represents the size of the peer effect of the enhancement of UGDE in this city on that in neighboring cities. The spatial panel autoregressive model constructed in this paper is as follows:

3 $$ UGDE_{it} = \alpha + \gamma W_{ij} UGDE_{jt} + \theta X_{it} + \mu_{i} + \lambda_{t} + \varepsilon_{it} $$

where UGDE represents the urban green development efficiency. *γ* is the spatial autoregressive coefficient, that is, the coefficient of peer effect, if *γ* is greater than 0, it indicates that the increase of UGDE in this city has a positive peer effect on that in neighboring cities; if *γ* is less than 0, there is a negative peer effect. *t* represents the year, *α* is the constant term, and *θ* represents the coefficient of the control variables. *X* is a series of control variables that affect UGDE, including foreign direct investment (*FDI*), industrial structure (*IS*), government investment in science, technology and education (*STE*) and informatization level (*IN*). Foreign direct investment is represented by the natural logarithm of the amount of foreign capital actually utilized; industrial structure is measured by the ratio of the added value of the secondary industry to GDP; government investment in science, technology and education is measured by the proportion of science, technology and education expenditure in the general public budget; informatization level is represented by the natural logarithm of the number of Internet broadband users. *ε*_*it*_ represents the random disturbance term, and *μ*_*i*_ and *λ*_*t*_ represent the spatial fixed effect and temporal fixed effect, respectively.

*W* is the spatial weight matrix. A suitable spatial weight matrix can accurately reflect the interaction between spatial units. In this paper, the reciprocal of the geographic distance between cities is used to construct the spatial weight matrix of geographic distance *W*^1^, and the formula is as follows:

4 $$ W_{{_{ij} }}^{1} = \left\{ {\begin{array}{*{20}l} {\frac{1}{{d_{ij} }},} \hfill & {if \, i \ne j} \hfill \\ {0,} \hfill & {if \, i = j} \hfill \\ \end{array} } \right. $$

where* d*_*ij*_ is the distance between city* i* and city *j* calculated from the latitude and longitude data of the cities. In this paper, in order to eliminate the impact of the dimension and numerical range of the data in the matrix, the spatial weight matrix of geographic distance is row-normalized.

In addition, in the robustness test part, this paper selects the reciprocal of the square of the geographical distance between cities to construct the spatial weight matrix *W*^2^, which is used to replace the spatial weight matrix of geographical distance to carry out the robustness test, the formula is as follows:

5 $$ W_{{_{ij} }}^{2} = \left\{ {\begin{array}{*{20}l} {\frac{1}{{d_{ij}^{2} }},} \hfill & {if \, i \ne j} \hfill \\ {0,} \hfill & {if \, i = j} \hfill \\ \end{array} } \right. $$

In order to further study the peer effects of UGDE, this paper refines the analysis of spatial interaction between cities, and investigates the peer effects of UGDE between cities of different grades. This paper first assumes that the spatial lag term of UGDE is *UGDElag it* = *W*_*ij*_*UGDE*_*it*_, and then, referring to the Notice of the State Council on Adjusting the Criteria for Urban Scale Division issued by the China’s government in 2014, divides prefecture-level and above cities in China into large cities, medium cities, and small cities, which are represented by symbols L, M, and S, respectively. Among them, cities with an urban population of more than 1 million are large cities, cities with an urban population of more than 500,000 to less than 1 million are medium cities, and cities with an urban population of less than 500,000 are small cities. Drawing on the relevant research^37,38^, this paper decomposes *UGDElag it* into:

6 $$ UGDE_{it}^{lag} = UGDE_{it}^{L} + UGDE_{it}^{M} + UGDE_{it}^{S} $$

Finally, this paper sets three dummy variables L, M and S to identify three types of cities, which are respectively multiplied with Formula (6), and then, obtains nine spatial lag terms, including *UGDEL itL*, *UGDEL itM*, *UGDEL itS*, *UGDEM itL*, *UGDEM itM*, *UGDEM itS*, *UGDES itL*, *UGDES itM*, *UGDES itS*. Thus, the peer effects between cities of different grades can be estimated^39^. Among them, *UGDEL itL*, *UGDEL itM* and *UGDEL itS* are respectively used to study the peer effects of large cities on three types of cities; *UGDEM itL*, *UGDEM itM*, *UGDEM itS* are respectively used to study the peer effects of medium cities on three types of cities; *UGDES itL*, *UGDES itM*, *UGDES itS* are respectively used to study the peer effects of small cities on three types of cities. Then Eq. (3) is transformed into:

7 $$ \begin{aligned} UGDE_{it} & = \alpha + \gamma_{1} UGDE_{it}^{L} L + \gamma_{2} UGDE_{it}^{L} M + \gamma_{3} UGDE_{it}^{L} S + \gamma_{4} UGDE_{it}^{M} L \\ & \quad + \gamma_{5} UGDE_{it}^{M} M + \gamma_{6} UGDE_{it}^{M} S + \gamma_{7} UGDE_{it}^{S} L + \gamma_{8} UGDE_{it}^{S} M \\ & \quad + \gamma_{9} UGDE_{it}^{S} S + \theta X_{it} + \mu_{i} + \lambda_{t} + \varepsilon_{it} \\ \end{aligned} $$

where *γ*_1_…*γ*_9_ are the corresponding coefficients of the peer effects.

### Kernel density estimation

Kernel density estimation is a non-parametric estimation method to estimate the probability density function, which does not need to make any parameter model assumptions, only studies the data characteristics from the data itself, and uses continuous density curve to describe the distribution form and dynamic evolution trend of random variables. It is a common method to study the dynamic evolution characteristics of random variables.

Suppose the random variables *X*_*1*_,* X*_*2*_,* X*_*3*_, …,* X*_*n*_ are independent and equally distributed, and the kernel density estimation formula is shown in Eq. (8).

8 $$ \begin{aligned} f_{n} \left( x \right) & = \frac{{F_{n} \left( {x + h} \right) - F_{n} \left( {x - h} \right)}}{2h} \\ & = \frac{1}{2h}\int\limits_{x - h}^{x + h} {dF_{n} \left( t \right)} \\ & = \int\limits_{ - \infty }^{ + \infty } {\frac{1}{h}K\left( {\frac{x - t}{h}} \right)dF_{n} \left( t \right)} \\ & = \frac{1}{nh}\sum\limits_{i = 1}^{n} K \left( {\frac{{x - X_{i} }}{h}} \right) \\ \end{aligned} $$

where *K* (⋅) is the kernel function, *x* is the mean, *n* is the number of samples, and *h* expresses the bandwidth.

The implementation form of kernel density estimation depends on the type of kernel function used, this paper uses Gaussian kernel function as the kernel function, whose expression is shown in Eq. (9).

9 $$ K\left( {\text{x}} \right) = \frac{1}{{\sqrt {2\pi } }}{\text{e}}^{{ - \frac{{x^{2} }}{2}}} $$

### Center of gravity and standard deviation ellipse model

#### Center of gravity model

The center of gravity model is used to describe the direction and distance of the center of gravity movement of certain attribute values in the region, this model has been widely used in existing literature, for example, Gai and Zhan studied the movement path of the center of gravity of marine eco-efficiency in China's coastal provinces and found that the spatial distribution range of the center of gravity of the efficiency showed a trend of expanding and then shrinking^40^. This paper uses the center of gravity model to describe the spatial change trajectory of UGDE and analyze its spatial evolution characteristics. The center of gravity coordinates are shown in Eq. (10).

10 $$ \left( {X,Y} \right) = \left( {\frac{{\sum\nolimits_{i = 1}^{n} {m_{i} x_{i} } }}{{\sum\nolimits_{i = 1}^{n} {m_{i} } }},\frac{{\sum\nolimits_{i = 1}^{n} {m_{i} y_{i} } }}{{\sum\nolimits_{i = 1}^{n} {m_{i} } }}} \right) $$

where (*X, Y*) represents the center of gravity coordinates of urban green development efficiency; (*x*_*i*_, *y*_i_) is the geographical coordinate of city *i*, *m*_*i*_ is the weight of city *i* and measured by UGDE of city *i*.

#### Standard deviation ellipse model

The standard deviation ellipse model is one of the classical methods used to describe the spatial distribution characteristics of certain attribute values, which can quantitatively explain the overall change of spatial morphology of the attribute values from the global and spatial perspectives. It includes three basic parameters, namely, the rotation angle, the long axis standard deviation and the short axis standard deviation. The rotation angle represents the main direction of the distribution of the research object, that is, the angle formed clockwise from the direction of true north to the long axis of the ellipse. The standard deviation of long axis and the standard deviation of short axis represent the dispersion degree of the research object in the direction of long axis and short axis respectively. In this paper, the standard deviation ellipse model is used to describe the spatial characteristics of UGDE and analyze its spatial evolution law. The calculation method of rotation angle, long axis standard deviation and short axis standard deviation are shown respectively in Eqs. (11)–(13)

11 $$ \tan \theta = \frac{{\left( {\sum\nolimits_{i = 1}^{n} {m_{i}^{2} \overline{x}_{i}^{2} } - \sum\nolimits_{i = 1}^{n} {m_{i}^{2} \overline{y}_{i}^{2} } } \right) + \sqrt {\left( {\sum\nolimits_{i = 1}^{n} {m_{i}^{2} \overline{x}_{i}^{2} } - \sum\nolimits_{i = 1}^{n} {m_{i}^{2} \overline{y}_{i}^{2} } } \right)^{2} + 4\sum\nolimits_{i = 1}^{n} {m_{i}^{2} \overline{x}_{i}^{2} \overline{y}_{i}^{2} } } }}{{2m_{i}^{2} \overline{x}_{i} \overline{y}_{i} }} $$

12 $$ \delta_{x} = \sqrt {\frac{{\sum\nolimits_{i = 1}^{n} {\left( {m_{i} \overline{x}_{i} \cos \theta - m_{i} \overline{y}_{i} \sin \theta } \right)^{2} } }}{{\sum\nolimits_{i = 1}^{n} {m_{i}^{2} } }}} $$

13 $$ \delta_{y} = \sqrt {\frac{{\sum\nolimits_{i = 1}^{n} {\left( {m_{i} \overline{x}_{i} \sin \theta - m_{i} \overline{y}_{i} \cos \theta } \right)^{2} } }}{{\sum\nolimits_{i = 1}^{n} {m_{i}^{2} } }}} $$

where *θ* is the rotation angle of the standard deviation ellipse, $(\overline{x}_{i} ,\overline{y}_{i} )$ is the relative coordinate of the distance between the city *i* and the center of the ellipse, *m*_*i*_ is the weight of city *i* and measured by UGDE of city *i*. *δ*_*x*_ expresses the long axis standard deviation, and *δ*_*y*_ represents the short axis standard deviation.

### Data sources

This paper selects the panel data of 283 cities at prefecture level and above in China from 2003 to 2017 as the research sample, which does not include Hong Kong Special Administrative Region, Macau Special Administrative Region, and cities in Taiwan Province. Due to the serious lack of data in some years, this paper excludes some cities in Tibet, Xinjiang, Hainan, Guizhou, Qinghai and other provinces, and the final number of sample cities is 283. For missing data, this paper uses interpolation to supplement. The original data comes from Urban Statistical Yearbook in China, Urban Construction Statistical Yearbook in China and the provincial statistical yearbooks. In order to avoid the bias of the estimation results caused by outliers, this paper carries out 1% double-tailed processing for all variables. The descriptive statistics of each variable are listed in Table 1.

**Table 1:** The descriptive statistics.

Variable	Obs	Mean	Std. Dev	Min	Max
UGDE	4245	0.532	0.428	0.077	9.575
FDI	4245	11.34783	2.245	0	16.835
IS	4245	0.484	0.110	0.027	0.910
STE	4245	0.197	0.047	0.006	0.497
IN	4245	12.507	1.255	5.466	17.762

## Results

### Analysis of temporal variation characteristics of UGDE

**Figure 1:**
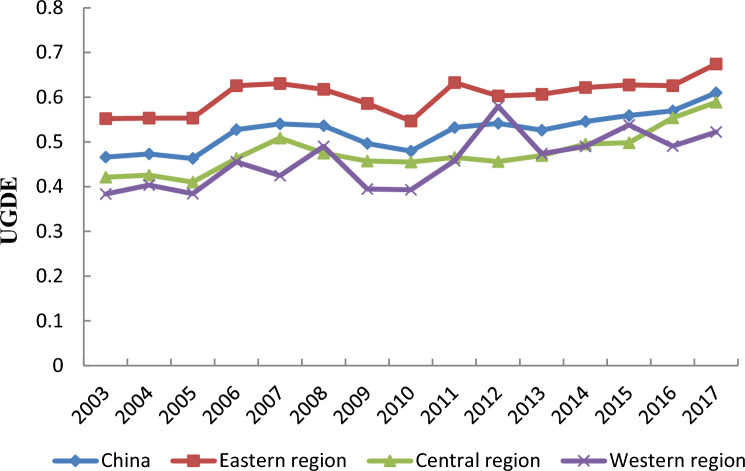
Change trends of average UGDE in China from 2003 to 2017.

Based on SE-SBM model, this paper uses Max-DEA software to measure the efficiency of urban green development in China. The change trend of average UGDE in China from 2003 to 2017 is depicted in Fig. 1. Across the country, the average UGDE increased from 0.47 in 2003 to 0.61 in 2017, with a cumulative growth rate of 29.79% and an average annual growth rate of 1.99%, showing a rising trend on the whole, indicating that China’s cities was committed to coordinating the promotion of economic development and environmental protection, addressing the various factors that hinder green development, and gradually promoting the optimal use of production factors such as energy and resources. This may be because China has implemented a series of green and low-carbon development and environmental protection policies and measures in the past decade or two, which have driven the development of related green industries and promoted the improvement of environmental quality. In the eastern region, the average UGDE rose from 0.55 in 2003 to 0.67 in 2017; in the central region, the average UGDE increased from 0.42 in 2003 to 0.59 in 2017; In the western region, the average UGDE rose from 0.38 in 2003 to 0.52 in 2017, growth was recorded in all regions. However, in the western region, the fluctuation was larger, possibly because the green development policy was incoherent, and the continuous improvement of UGDE could not be achieved. In the past years, the eastern region had the highest average UGDE, followed in most years by the central region and the lowest in the western region, it presented a gradually decreasing spatial distribution pattern from the eastern region to the western region, and the spatial difference was obvious. This may be because each region was at a different stage of economic development. In addition, with the exception of 2012, the average UGDE in the central and western regions was lower than the national level, and the central and western regions should promote UGDE to reach the national average level by improving green development policies and encouraging the development of green industries.

**Figure 2:**
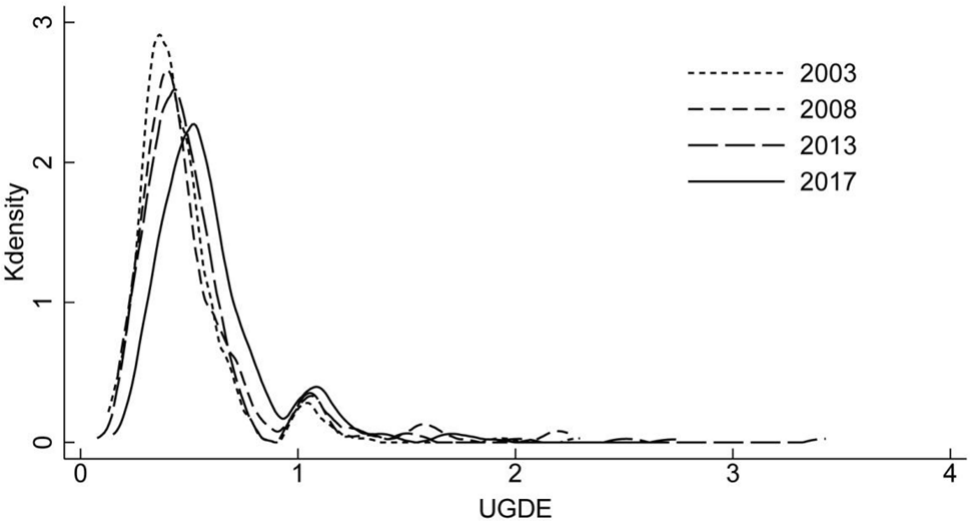
Kernel density curve of UGDE in China.

This paper estimates the kernel density of UGDE in China in 2003, 2008, 2013 and 2017 respectively, and the kernel density curve is shown in Fig. 2. From the perspective of curve position changes, the kernel density curves in 2008, 2013 and 2017 all moved to the right, indicating that China's UGDE presented a gradual upward trend, which is consistent with the results in Fig. 1, confirming that UGDE in China continued to improve, and the effect of economic green transformation is obvious. From the perspective of distribution pattern, the peak height of kernel density curve decreased and its width widened in 2008 compared with 2003, which means that the difference of UGDE between cities became larger. Compared with 2008, the peak height of the kernel density curve in 2013 continued to decrease, the width continued to widen, and the right tail lengthened, indicating that the spatial difference of UGDE continued to increase. Compared with 2013, the peak height of the kernel density curve continued to decrease and the width widened in 2017, indicating that the spatial difference of UGDE further expanded. In conclusion, the spatial difference of UGDE increased year by year, and the urban green development showed non-equilibrium.

### Analysis of spatial evolution characteristics of UGDE

#### Spatial distribution characteristics analysis

**Figure 3:**
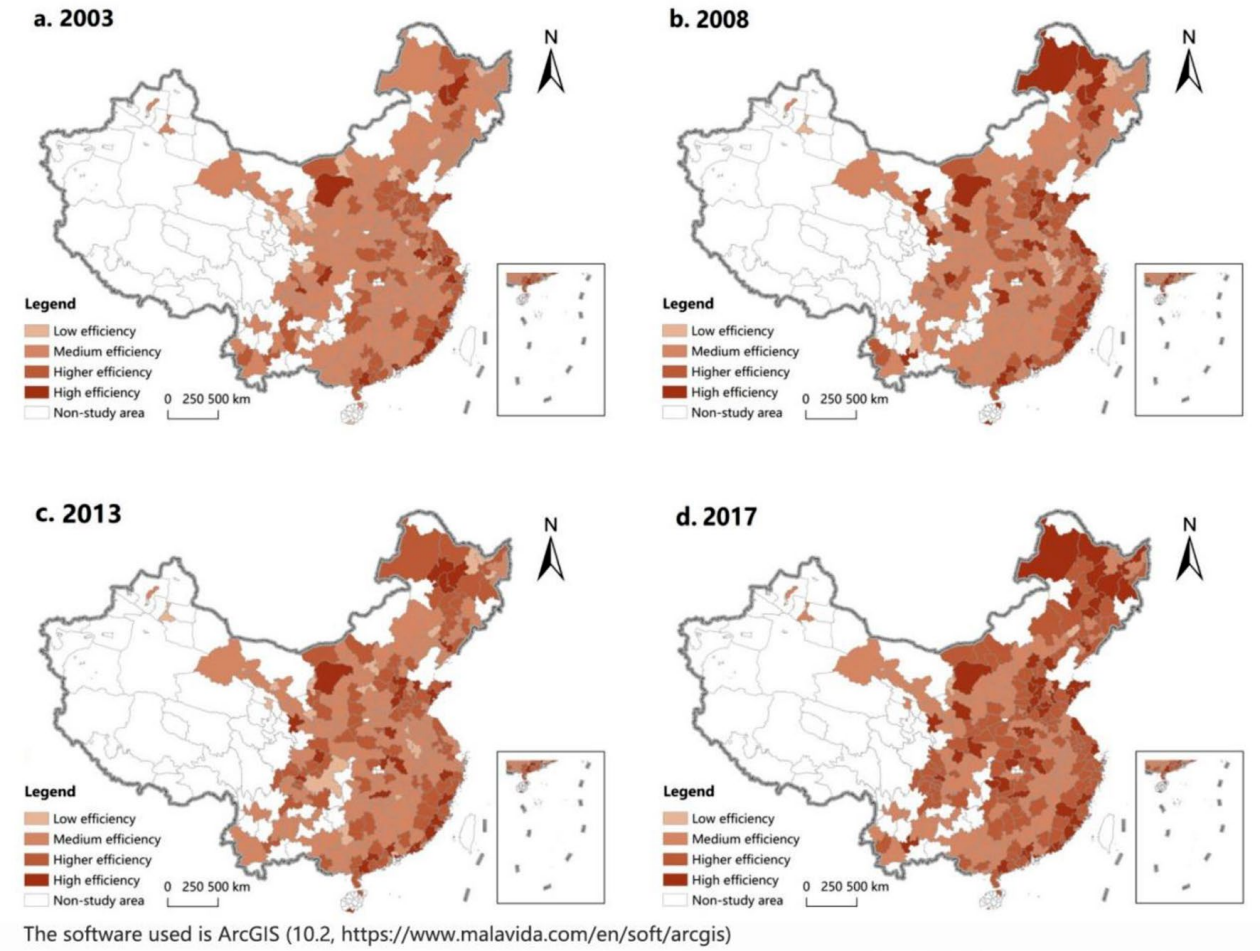
Spatial distribution of UGDE in China.

In order to intuitively reflect the spatial distribution characteristics of UGDE, based on the measurement results of SE-SBM model, this paper uses ArcGIS 10.2 software to draw the spatial distribution maps of UGDE in 2003, 2008, 2013 and 2017, as shown in Fig. 3. In this paper, efficiency values are divided into four levels from low to high. Cities with efficiency values between 0.00 and 0.25 are low-efficiency cities, cities with efficiency values between 0.25 and 0.5 are medium-efficiency cities, cities with efficiency values between 0.50 and 0.75 are high-efficiency cities, and cities with efficiency values greater than 0.75 are high-efficiency cities.

In 2003, the number of low-efficiency cities was 21, mainly in the central and western regions; the number of medium-efficiency cities was 180, mainly distributed in the eastern and central regions; the number of cities with higher efficiency and high efficiency were 63 and 19 respectively, mainly distributed in the eastern region, indicating that the performance of urban green development in the eastern region of China was remarkable, while that was poor in the central and western regions of China. In 2008, the number of low-efficiency and higher-efficiency cities remained basically unchanged, as did the main distribution areas; the number of medium-efficiency cities decreased to 160, and the main distribution areas remained unchanged; the number of high-efficiency cities doubled, reaching 38, the eastern region was still the main distribution region, but the number of high-efficiency cities in the central region increased significantly, from 4 in 2003 to 10, indicating that the central region has gradually changed its extensive economic development mode and made some achievements in urban green development at this stage. In 2013, the number of low-efficiency cities remained basically unchanged; the number of medium-efficiency cities continued to decrease to 150, and the main distribution area remained unchanged; the number of higher-efficiency cities increased to 83, the eastern region was still the main distribution region, but the number of higher-efficiency cities in the western region increased significantly, from 7 in 2008 to 16; however, the number of high-efficiency cities decreased, leaving only 29. In conclusion, urban green development in China did not make significant progress at this stage. In 2017, the number of low-efficiency cities significantly reduced to four; the number of medium-efficiency cities also fell sharply to 107, but the main decline areas was in the eastern and central regions; the number of higher-efficiency cities continued to increase to 117, with the main distribution areas expanding to the central region; the number of high-efficiency cities increased significantly to 56, and the main distribution areas also expanded to the central region, indicating that the level of urban green development in eastern and central China significantly enhanced at this stage, and the quality of economic development remarkably improved.

In short, during the study period, the number of low-efficiency cities and medium-efficiency cities continued to decrease, while the number of higher-efficiency cities and high-efficiency cities continued to increase, and China achieved remarkable results in the urban green development. The eastern region has always been one of the main distribution areas of higher-efficiency cities and high-efficiency cities; in the central region, UGDE in most cities improved significantly, the central region became one of the main distribution areas of higher-efficiency cities and high-efficiency cities; in the western region, although some success has been achieved in the urban green development, UGDE has always lagged behind that in the eastern and central regions.

#### Center of gravity migration and standard deviation ellipse analysis

**Figure 4:**
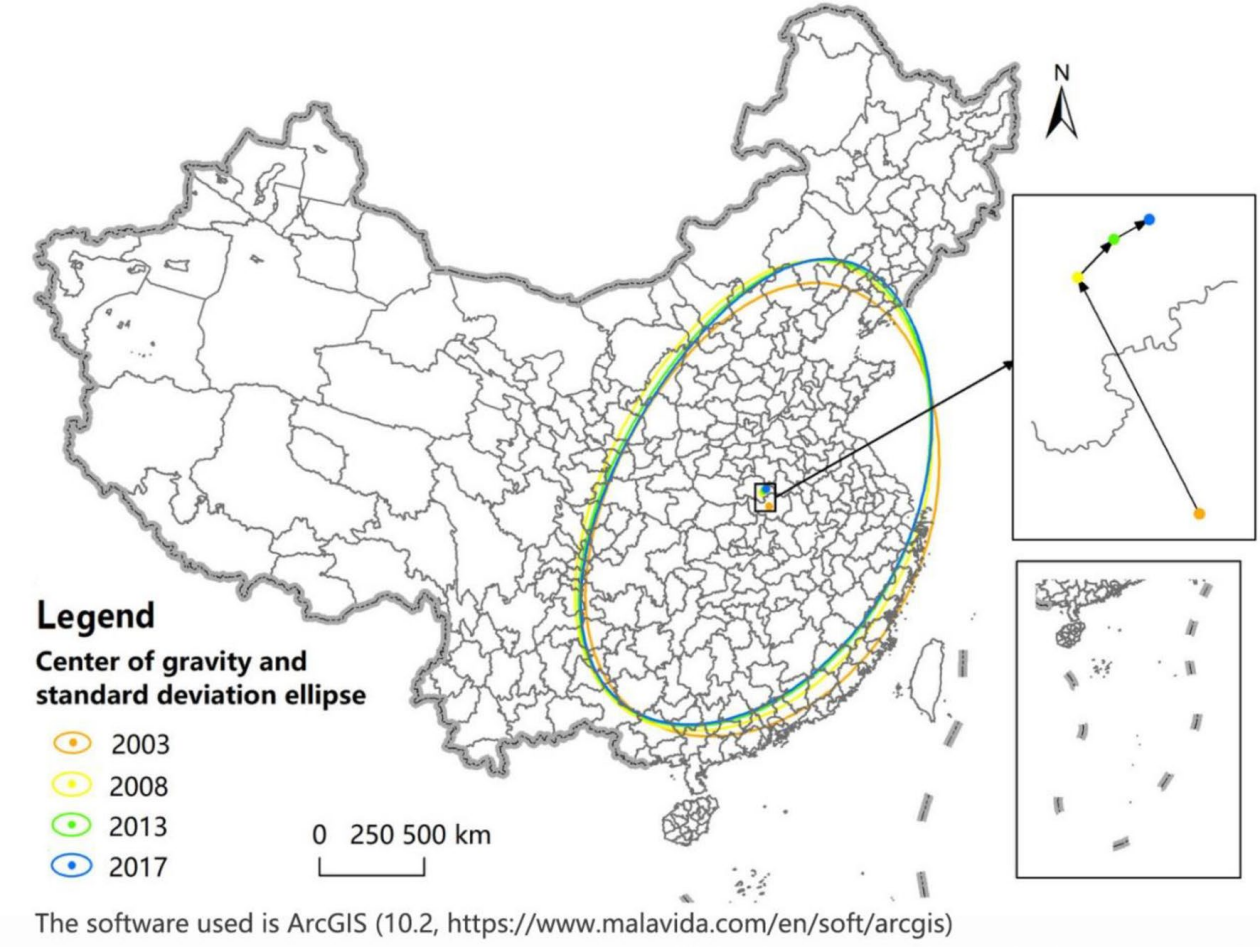
Center of gravity migration and standard deviation ellipse distribution of UGDE in China.

Based on the center of gravity model, the ArcGIS 10.2 software is used in this paper to calculate the center of gravity position of UGDE in China and the distance and direction of its migration in various periods, and to draw the center of gravity migration trajectory diagram, as shown in Fig. 4. From 2003 to 2017, the center of gravity of UGDE was located in Henan Province, China, with a total displacement of 100.07 km, showing a trend of northwest migration in general. From each stage, from 2003 to 2008, the center of gravity moved 74.08 km to the northwest; from 2008 to 2013, it shifted 14.64 km to the northeast; from 2013 to 2017, it continued to shift 11.35 km to the northeast.

Based on the standard deviation ellipse model, this paper uses the ArcGIS 10.2 software to calculate and identify the parameters of the standard deviation ellipse of UGDE in China in various periods, including the rotation angle, the standard deviation of long axis and the standard deviation of short axis, and draws the variation trend chart of the standard deviation ellipse, as shown in Fig. 4. From 2003 to 2017, *θ* showed a trend of decreasing first and then increasing, but the overall change was little, and the ellipse distributed in the northeast-to-southwest direction. The ellipse area first increased and then decreased, increasing by 85,324.80 km^2^ from 2003 to 2008, and decreasing by 146,495.61 km^2^ from 2008 to 2017. From the distribution range of the ellipse, the standard deviation of long axis contracted year by year, from 845.125 km in 2003 to 792.05 km in 2017, reducing by 53.08 km; the standard deviation of short axis expanded year by year, from 1223.10 km in 2003 to 1293.96 km in 2017, with an increase of 70.86 km, indicating that UGDE contracted in the east–west direction and expanded in the north–south direction, showing a spatial dispersion trend.

#### Regional spatial dynamics analysis

**Figure 5:**
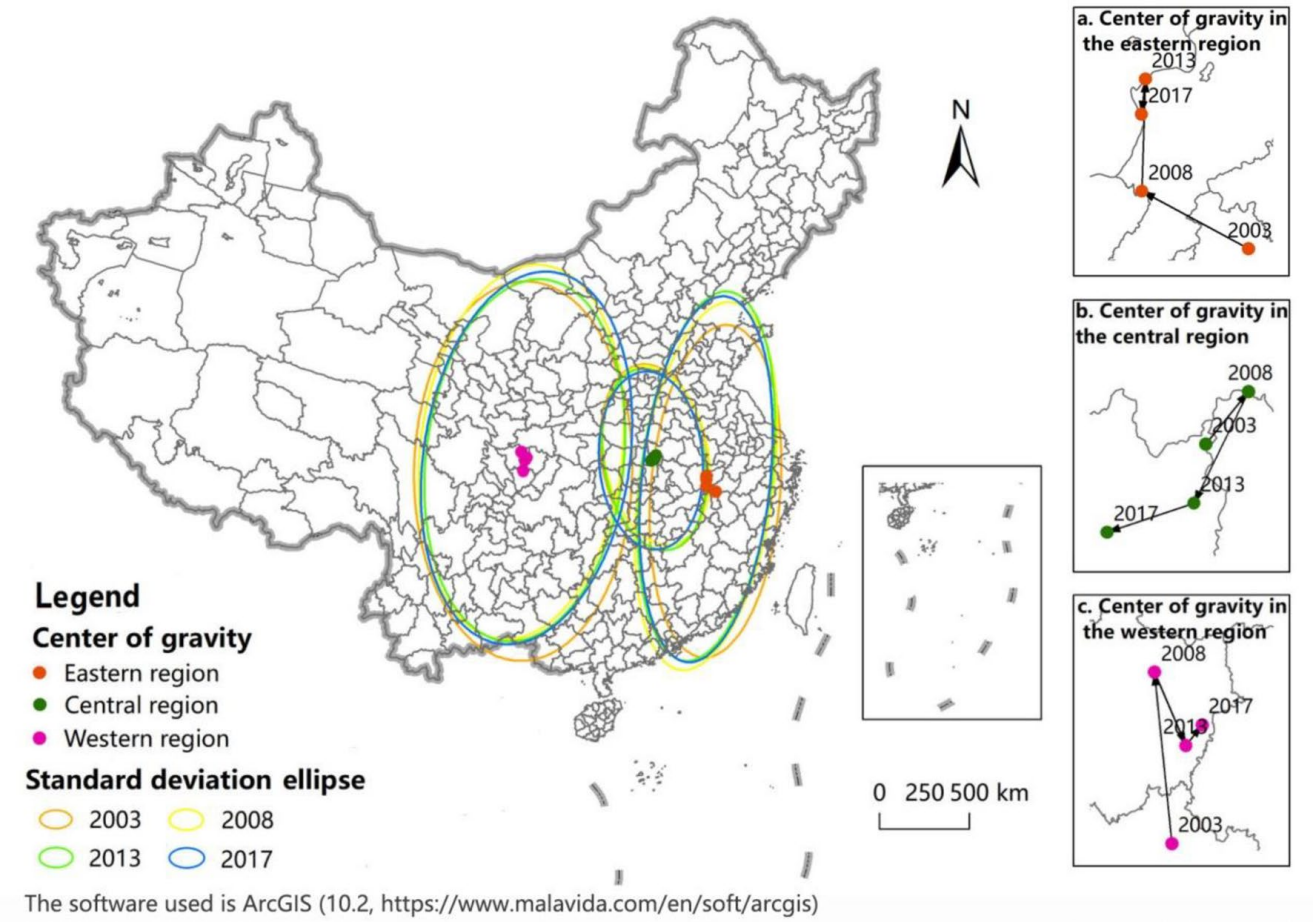
Center of gravity migration and standard deviation ellipse distribution of UGDE in different regions of China.

From the above analysis, it can be seen that the spatial dispersion trend of UGDE is obvious. In order to further study the spatial evolution law of UGDE, this paper analyzes the internal spatial evolution characteristics in the eastern, central, western regions of China, and the center of gravity migration trajectory and the change trend of standard deviation ellipse in each region is shown in Fig. 5, the ArcGIS 10.2 software is used to calculate and draw. During the study period, in the eastern region of China, the center of gravity migrated to the northwest, and the ellipse distributed from northeast to southwest. The standard deviation of long axis did not change much, and the standard deviation of short axis first increased and then decreased, with an overall increase of 95.34 km, and the ellipse area increased by 98,389.06 km^2^, representing that the spatial distribution of UGDE in the eastern region showed a dispersed trend. In the central region of China, the center of gravity migrated to the southwest, and the ellipse distributed from northwest to southeast. The standard deviation of long axis did not change much, while the standard deviation of short axis decreased by 25.53 km, and the ellipse area contracted by 31,976.40 km^2^, which meant that the spatial agglomeration characteristics of UGDE continuously strengthened. In the western region of China, the center of gravity migrated to the northeast, and the ellipse distributed northeast-southwest. The standard deviation of long axis decreased first and then increased, decreasing by 33.21 km on the whole; the standard deviation of short axis changed little, and the ellipse area decreased by 130,642.79 km^2^, which indicated that the spatial distribution of UGDE in the western region presented a clustering trend.

### Econometric results analysis

In this paper, the maximum likelihood method is used to estimate Eq. (7). In order to ensure the robustness of the estimation results, control variables are gradually added during the estimation, the estimation results are shown in Table 2. It can be seen that the estimated results of each column in Table 2 have little difference, which represents that the estimated results of the model are robust. Column 4 represents the estimated results with all control variables included in the model, this paper focuses on the analysis of the results in this column.

**Table 2:** Regression results of peer effects.

Variables	(1)	(2)	(3)	(4)
STE	0.083 (0.187)	0.087 (0.173)	0.092 (0.164)	0.087 (0.176)
IS		0.037 (0.104)	0.010 (0.082)	0.014 (0.101)
FDI			0.007 (0.005)	0.008 (0.005)
IN				− 0.003 (0.006)
Higher → Lower				
UGDE^L^M	2.061*** (0.498)	2.056*** (0.589)	2.004*** (0.626)	2.038*** (0.479)
UGDE^L^S	1.567** (0.683)	1.559** (0.656)	1.487** (0.687)	1.551** (0.630)
UGDE^M^S	1.745*** (0.625)	1.744*** (0.486)	1.630*** (0.545)	1.675*** (0.535)
Lower → Higher				
UGDE^M^L	2.229*** (0.498)	2.226*** (0.540)	2.080*** (0.605)	2.129*** (0.582)
UGDE^S^L	− 0.108 (0.287)	− 0.110 (0.304)	− 0.165 (0.292)	− 0.124 (0.294)
UGDE^S^M	0.683* (0.375)	0.678* (0.366)	0.644* (0.381)	0.673* (0.352)
The same grade				
UGDE^L^L	1.724*** (0.599)	1.747*** (0.659)	1.678*** (0.614)	1.715*** (0.605)
UGDE^M^M	0.535 (0.598)	0.551 (0.833)	0.421 (0.710)	0.482 (0.793)
UGDE^S^S	0.345 (0.233)	0.351 (0.298)	0.339 (0.280)	0.364 (0.325)
Constant	− 0.020 (0.072)	− 0.039 (0.106)	− 0.071 (0.094)	− 0.060 (0.111)
Log L	1218.000	1218.304	1221.191	1221.505
N	4245	4245	4245	4245

The values in the parentheses are standard errors; Log L represents Log likelihood.

***p < 0.01, **p < 0.05, *p < 0.1

From the perspective of the peer effects of higher-grade cities on lower-grade cities, the coefficient of *UGDE*^*L*^*M* is 2.038, significant at 1% level, which confirms that the improvement of UGDE in large cities has a driving effect on that in neighboring medium cities through the positive peer effect. The coefficient of *UGDE*^*L*^*S* is 1.551, significant at 5% level, which means that the increase of UGDE in large cities has a promoting effect on that in neighboring small cities through the positive peer effect. The coefficient of *UGDE*^*M*^*S* is 1.675, which is significant at 1% level, indicating that the growth of UGDE in medium cities has a driving effect on that in neighboring small cities through the positive peer effect. From the perspective of the peer effects of lower-grade cities on higher-grade cities, the coefficient of *UGDE*^*M*^*L* is 2.129 and significant, which represents that the improvement of UGDE in medium cities has a positive peer effect on that in neighboring large cities. The coefficient of *UGDE*^*S*^*L* is − 0.124, which is not significant, indicating that the peer effect of small cities on neighboring large cities is not significant. The regression coefficient of *UGDE*^*S*^*M* is 0.673 and significant, which confirms that the growth of UGDE in small cities has a positive peer effect on that in neighboring medium cities. From the point of view of the peer effects between cities of the same grade, the coefficient of *UGDE*^*L*^*L* is 1.715, which is significant at 1% level, showing that UGDE promotes each other between large cities through the positive peer effect. The coefficients of *UGDE*^*M*^*M* and *UGDE*^*S*^*S* are not significant, which means that the peer effects between medium cities and between small cities are not significant.

### Robustness test

In order to verify the robustness of the regression results, this paper uses the reciprocal of the square of the geographical distance between cities to construct a new spatial weight matrix for robustness test, the regression results are shown in column 1 of Table 3. It can be seen from the results that except the coefficient of *UGDE*^*M*^*L* is not significant, the rest of the results are consistent with the results in the benchmark model. The robustness test was also carried out by adding control variables and removing samples of municipalities, the results are shown in columns 2 and 3 of Table 3 respectively, which are consistent with those in column 1. Therefore, robustness test confirms that the conclusion of the benchmark model is robust.

**Table 3:** Robustness test results.

Variables	(1) Change spatial weight matrix	(2) Increase an explanatory variable	(3) Exclude the sample of province-level municipality
STE	0.126 (0.158)	0.057 (0.162)	0.074 (0.137)
IS	− 0.070 (0.081)	0.008 (0.094)	0.033 (0.093)
FDI	0.009* (0.005)	0.007 (0.005)	0.008 (0.005)
IN	0.011** (0.005)	− 0.005 (0.006)	− 0.002 (0.005)
POP		1.446** (0.588)	
Higher → Lower			
UGDE^L^M	0.750*** (0.178)	2.058*** (0.597)	2.028*** (0.601)
UGDE^L^S	0.768** (0.384)	1.614** (0.725)	1.521** (0.702)
UGDE^M^S	0.797*** (0.213)	1.579*** (0.611)	1.635*** (0.528)
Lower → Higher			
UGDE^M^L	− 0.048 (0.153)	− 0.133 (0.335)	− 0.089 (0.351)
UGDE^S^L	0.423*** (0.146)	0.696* (0.395)	0.634* (0.365)
UGDE^S^M	1.108*** (0.297)	1.999*** (0.652)	2.137*** (0.497)
The same grade			
UGDE^L^L	0.794*** (0.200)	1.735*** (0.566)	1.495** (0.600)
UGDE^M^M	0.473 (0.291)	0.425 (0.763)	0.442 (0.949)
UGDE^S^S	0.422*** (0.105)	0.452 (0.298)	0.336 (0.295)
Constant	0.003 (0.091)	− 0.081 (0.104)	− 0.060 (0.112)
Log L	1208.412	1226.502	1215.351
N	4245	4245	4185

The values in the parentheses are standard errors; Log L represents Log likelihood.

***p < 0.01, **p < 0.05, *p < 0.1

### Heterogeneity analysis

China has a vast territory, and different regions are at different stages of development, and there are differences in resource endowment, energy structure and industrial structure, which may lead to the heterogeneity of peer effects of UGDE in different regions. According to the geographical location of cities, China’s cities are divided into eastern cities, central cities and western cities, which are used as samples for regression. The heterogeneity test results are described in Table 4, columns 1, 2 and 3 represent the estimated results of the eastern, central and western regions respectively. The results show that in the eastern region, the growth of UGDE in large cities has a positive peer effect on that in neighboring medium cities; the improvement of UGDE in medium cities has a promoting effect on that in neighboring small cities through the positive peer effect; the increase of UGDE in small cities has a positive peer effect on that in neighboring large and medium cities; UGDE promotes each other between large cities through the positive peer effect. In the central region, the increase of UGDE in large cities has a positive peer effect on that in neighboring medium and small cities; the increase of UGDE in small cities has a driving effect on that in neighboring medium cities through the positive peer effect. In the western region, UGDE promotes each other between large cities and between small cities through the positive peer effect. In conclusion, the peer effects of UGDE have regional heterogeneity, showing different characteristics in eastern, central and western China.

**Table 4:** Heterogeneity test results.

Variables	(1) Eastern region	(2) Central region	(3) Western region
STE	0.349 (0.351)	− 0.162 (0.226)	− 0.022 (0.310)
IS	− 0.261* (0.135)	− 0.062 (0.117)	0.430* (0.241)
FDI	0.009 (0.007)	0.002 (0.006)	0.010 (0.011)
IN	− 0.010 (0.009)	0.005 (0.010)	− 0.014* (0.008)
Higher → Lower			
UGDE^L^M	1.412** (0.697)	2.080*** (0.648)	2.976 (1.995)
UGDE^L^S	1.428 (1.135)	2.335*** (0.737)	1.709 (1.574)
UGDE^M^S	2.602*** (0.882)	0.959 (0.750)	0.378 (0.951)
Lower → Higher			
UGDE^M^L	0.181 (0.472)	− 0.579 (0.603)	0.466 (0.467)
UGDE^S^L	1.495* (0.897)	− 0.001 (0.519)	0.779 (0.746)
UGDE^S^M	1.939*** (0.696)	2.980*** (1.105)	0.634 (0.977)
The same grade			
UGDE^L^L	1.470* (0.882)	0.903 (0.877)	2.459* (1.422)
UGDE^M^M	0.018 (1.005)	1.000 (0.828)	− 0.342 (1.139)
UGDE^S^S	− 0.261 (0.591)	0.190 (0.323)	1.201*** (0.462)
Constant	0.123 (0.172)	0.021 (0.131)	− 0.183 (0.201)
Log L	386.718	770.994	167.130
N	1725	1635	885

The values in the parentheses are standard errors; Log L represents Log likelihood.

***p < 0.01, **p < 0.05, *p < 0.1

## Discussion

### Peer effects of UGDE between cities of different grades

The research conclusions indicate that UGDE has significant peer effects between cities of different grades, which is consistent with the research conclusions of Guo et al. and Zhou et al. ^19,20^, who concluded that UGDE has a significant spatial spillover effect, and the peer effect is essentially a spatial spillover effect, but its research focuses on the strategic interaction among economic agents^14^. In other words, in terms of UGDE, there is an obvious spatial interaction among large cities, medium cities and small cities, which promotes the improvement of UGDE in surrounding cities, and plays an important role in the regional green coordinated development. Therefore, the internal driving force of regional green coordinated development lies in the existence of peer effects between cities of different grades, which promotes regional green coordinated development.

The empirical results show that from the perspective of the peer effects of higher-grade cities on lower-grade cities, the improvement of UGDE in large cities has a driving effect on that in neighboring medium cities and small cities through the positive peer effect; the growth of UGDE in medium cities has a promoting effect on that in neighboring small cities, that is, the increase of UGDE in higher-grade cities has a promoting effect on that in neighboring lower-grade cities through the positive peer effects. This may be because there is a top-down green development radiation driving effect among cities in China. The higher-grade cities represented by large cities and medium cities play an exemplary and leading role in green development, and small cities will adopt a learning imitation strategy to formulate green development policies similar to those of neighboring large cities and medium cities, thus promoting the green development of the city and improving its efficiency. From the perspective of the peer effects of lower-grade cities on higher-grade cities, the improvement of UGDE in medium cities has a positive peer effect on that in neighboring large cities; the growth of UGDE in small cities has a positive peer effect on that in neighboring medium cities. This may be because higher-grade cities have a certain sense of crisis when faced with the achievements of lower-grade cities in green development, they will pay more attention to urban green development, and then adopt more green development policies and measures to promote urban green development. From the point of view of the peer effects between cities of the same grade, UGDE promotes each other between large cities through the positive peer effect, while the peer effects between medium cities and between small cities are not significant. This may be because large cities are more sensitive to the performance and policies of similar cities in green development, will promote urban green development through competitive imitation strategies.

### Policy implications

#### Establish a collaborative mechanism for green development between neighboring cities

Establishing a collaborative mechanism for green development between neighboring cities is one of the important ways to improve the efficiency of urban green development, and it is also a necessary measure for regional cities to cope with resource and environmental challenges and build green development highlands. First, break the flow restrictions of green production factors, green technology barriers, and green product market barriers among neighboring cities, realize the free flow of green production factors, efficient allocation of green resources, and correlation and supporting of green industries among neighboring cities, jointly promote green technology innovation, promote the coordinated development of regional green industries, and better play the peer effect of green development efficiency among neighboring cities. Second, based on the principle of cost sharing and benefit sharing, strengthen cooperation in pollution prevention and control, joint law enforcement, unified standards and other fields, strengthen the concept of regional integrity, and gradually form a long-term trans-regional environmental collaborative governance mechanism with integrated measures, complementary advantages and mutual benefit. At the same time, jointly promote the fight against " greenwashing" behavior, and create a good environment for green development. Third, build an environmental information resource sharing and interconnection platform, establish an automatic monitoring and abnormal alarm mechanism for pollution emissions of key polluters, as well as a monitoring data sharing and release mechanism for key pollution sources, strengthen pollution source tracking and analysis, and promote real-time sharing of environmental monitoring information, business information and government information, grasp the environmental pollution situation in time through automatic early warning of environmental quality.

#### Formulate green development plans for urban agglomerations

In order to better exert the peer effects of UGDE between cities of different grades, it is necessary to formulate the green development plans of urban agglomeration as a whole and clarify the green development goals, which is an important measure to realize the sustainable development of urban agglomeration. Firstly, strengthen the green transformation of traditional industries in urban agglomerations, eliminate backward and inefficient production capacity, limit the development of highly polluting and energy-consuming industries, update technological processes, improve energy efficiency, reduce pollutant emissions, and comprehensively promote clean production. Secondly, focus on building new engines of industrial development with low resource and energy consumption, high economic added value, and low environmental pollution in urban agglomerations, develop strategic emerging industries such as new energy vehicles, intelligent manufacturing, and new materials, accelerate the development of energy-saving and environmental protection industries, promote innovation in green service models, and promote the transformation and upgrading of industrial structure through the development of green industries. Thirdly, vigorously develop a circular economy in urban agglomerations, strengthen the comprehensive utilization of resources and the coordinated disposal of urban waste, guide enterprises to use non-toxic and harmless environmentally friendly raw materials, promote product design schemes that are easy to disassemble, classify and recycle, jointly construct the waste recycling facilities among cities, and improve the waste recycling networks, coordinate the construction of waste recycling outlets and household waste sorting outlets, and build a recycling system for waste materials.

#### Reform the assessment system of local government officials

By reforming the assessment system of local government officials, cultivating the concept of green achievements, advocating more diversified and more public value assessment of officials' performance, building a green development assessment system for local government officials, giving better play to the "baton" role of the assessment system, and driving local government officials to promote economic development guided by green development. First of all, to ensure that the assessment indicators can reflect the green development of the city and improve the quality of the city, the green development assessment indicators for officials should be set from the aspects of resource utilization, environmental governance, environmental quality, ecological protection, growth quality, green life, and public satisfaction. Secondly, pay attention to the fine design of green development indicators, promote the coordinated green development with other cities as one of the assessment indicators, and promote the cooperation between urban governments in green development, avoid disorderly competition, and jointly promote the improvement of regional green development efficiency. Finally, strengthen the periodic assessment of local government officials, such as mid-term assessment, so as to reserve time for policy adjustment and response to emergencies, and ensure the realization of green development goals.

### Limitations and future research directions

Based on the measurement of UGDE, this paper studies the spatial–temporal evolution characteristics of UGDE and the peer effects of UGDE between cities of different grades. It is undeniable that the research in this paper has some limitations. In the measurement of UGDE, because some data are difficult to obtain, the construction of the index system is still lacking, as a result, the accuracy of measurement results is not high enough. In terms of research content, this paper only studies the spatial–temporal evolution characteristics and the peer effects of UGDE, future studies can further explore the specific mechanism and influencing factors causing the peer effects, so as to form a complete research system and provide a theoretical analysis framework for other scholars to study such issues. In addition, future research can also take a certain region as the research object, combined with the specific reality of the region, to further study the peer effects of UGDE, thus providing more valuable and practical reference for regional green development.

## Conclusions

Based on the panel data of 283 cities at prefecture level and above in China from 2003 to 2017, this paper measures urban green development efficiency and analyzes its spatial–temporal evolution characteristics. It is found that during the study period, in terms of temporal evolution, the average UGDE in China increased from 0.47 in 2003 to 0.61 in 2017, with a cumulative growth rate of 29.79% and an average annual growth rate of 1.99%, showing a rising trend in general. In terms of spatial evolution, the number of low-efficiency cities and medium-efficiency cities continued to decrease, while the number of higher-efficiency cities and high-efficiency cities continued to increase, and China achieved remarkable results in the urban green development. The eastern region has always been one of the main distribution areas of higher-efficiency cities and high-efficiency cities; in the central region, UGDE in most cities improved significantly, the central region became one of the main distribution areas of higher-efficiency cities and high-efficiency cities; in the western region, although some success has been achieved in the urban green development, UGDE has always lagged behind that in the eastern and central regions. In addition, based on the center of gravity model and the standard deviation ellipse model, it is found that the center of gravity of UGDE presented a trend of northwest migration in general, with a total displacement of 100.07 km; UGDE contracted in the east–west direction and expanded in the north–south direction, showing a spatial dispersion trend.

Based on the measurement results, this paper constructs a spatial econometric model to analyze the peer effects of UGDE between cities of different grades. The empirical results indicate that from the perspective of the peer effects of higher-grade cities on lower-grade cities, the improvement of UGDE in large cities has a driving effect on that in neighboring medium cities and small cities through the positive peer effect; the growth of UGDE in medium cities has a promoting effect on that in neighboring small cities through the positive peer effect. From the perspective of the peer effects of lower-grade cities on higher-grade cities, the improvement of UGDE in medium cities has a positive peer effect on that in neighboring large cities; the growth of UGDE in small cities has a positive peer effect on that in neighboring medium cities. From the point of view of the peer effects between cities of the same grade, UGDE promotes each other between large cities through the positive peer effect, while the peer effects between medium cities and between small cities are not significant. In addition, the peer effects of UGDE have regional heterogeneity, showing different characteristics in eastern, central and western China.

## Data Availability

The datasets used and/or analysed during the current study are available from the corresponding author on reasonable request.
